# Molecular Basis of KAT2A Selecting Acyl-CoA Cofactors for Histone Modifications

**DOI:** 10.34133/research.0109

**Published:** 2023-04-04

**Authors:** Sha Li, Nan Li, Jie He, Runxin Zhou, Zhimin Lu, Yizhi Jane Tao, Yusong R. Guo, Yugang Wang

**Affiliations:** ^1^ Department of Biochemistry and Molecular Biology, School of Basic Medicine, Tongji Medical College, Huazhong University of Science and Technology, Wuhan, Hubei 430030, China.; ^2^ Department of Neurosurgery, Union Hospital, Tongji Medical College, Huazhong University of Science and Technology, Wuhan 430022, China.; ^3^ Zhejiang University School of Medicine, Hangzhou, Zhejiang 310029, China.; ^4^ Department of Bioscience, Rice University, Houston, TX 77005, USA.; ^5^ Cell Architecture Research Center, Huazhong University of Science and Technology, Wuhan, Hubei 430030, China.

## Abstract

Emerging discoveries about undocumented acyltransferase activities of known histone acetyltransferases (HATs) advance our understandings in the regulation of histone modifications. However, the molecular basis of HATs selecting acyl coenzyme A (acyl-CoA) substrates for histone modification is less known. We here report that lysine acetyltransferase 2A (KAT2A) as an illustrative instance of HATs can selectively utilize acetyl-CoA, propionyl-CoA, butyryl-CoA, and succinyl-CoA to directly deposit 18 histone acylation hallmarks in nucleosome. By analyzing the co-crystal structures of the catalytic domain of KAT2A in complex with acetyl-CoA, propionyl-CoA, butyryl-CoA, malonyl-CoA, succinyl-CoA, and glutaryl-CoA, we conclude that the alternative substrate-binding pocket of KAT2A and the length and electrostatic features of the acyl chain cooperatively determine the selection of the acyl-CoA substrates by KAT2A. This study reveals the molecular basis underlying the pluripotency of HATs that selectively install acylation hallmarks in nucleosomes, which might serve as instrumental mechanism to precisely regulate histone acylation profiles in cells.

Lysine acylation is a group of lysine modifications chemically related to acetylation, including propionylation [1], butyrylation [1], malonylation [2], succinylation [2], glutarylation [3], crotonylation [4], and 2-hydroxyisobutyrylation [5]. They occur frequently on histones and contribute to the complexity about histone functions in regulating chromatin processes [6]. KAT2A is the first identified histone acetyltransferase (HAT) and succinyltransferase [7]. Additional studies further revealed the propionyltransferase and butyryltransferase activities of KAT2A [8], raising questions about the molecular basis underlying KAT2A-selecting acyl coenzyme A (acyl-CoA) substrates to manipulate histone acylation profiles. Here, we report that KAT2A can selectively utilize acetyl-CoA, propionyl-CoA, butyryl-CoA, and succinyl-CoA to directly deposit 18 histone acylation hallmarks in nucleosome. We co-crystallized the catalytic domain of KAT2A in complex with types of acyl-CoA. Structural analyses revealed that the length of the acyl chain and the alternative substrate pocket in the catalytic domain of KAT2A cooperatively determine KAT2A-selecting acyl-CoA for histone modification. This study provides an illustrative instance about the molecular basis underlying the pluripotency of HATs that directly install a broad spectrum of histone acylation hallmarks, emphasizing the complexity in the regulatory mechanisms of histone acylations.

## KAT2A is a Pluripotent Histone Acyltransferase

**Fig. 1.**
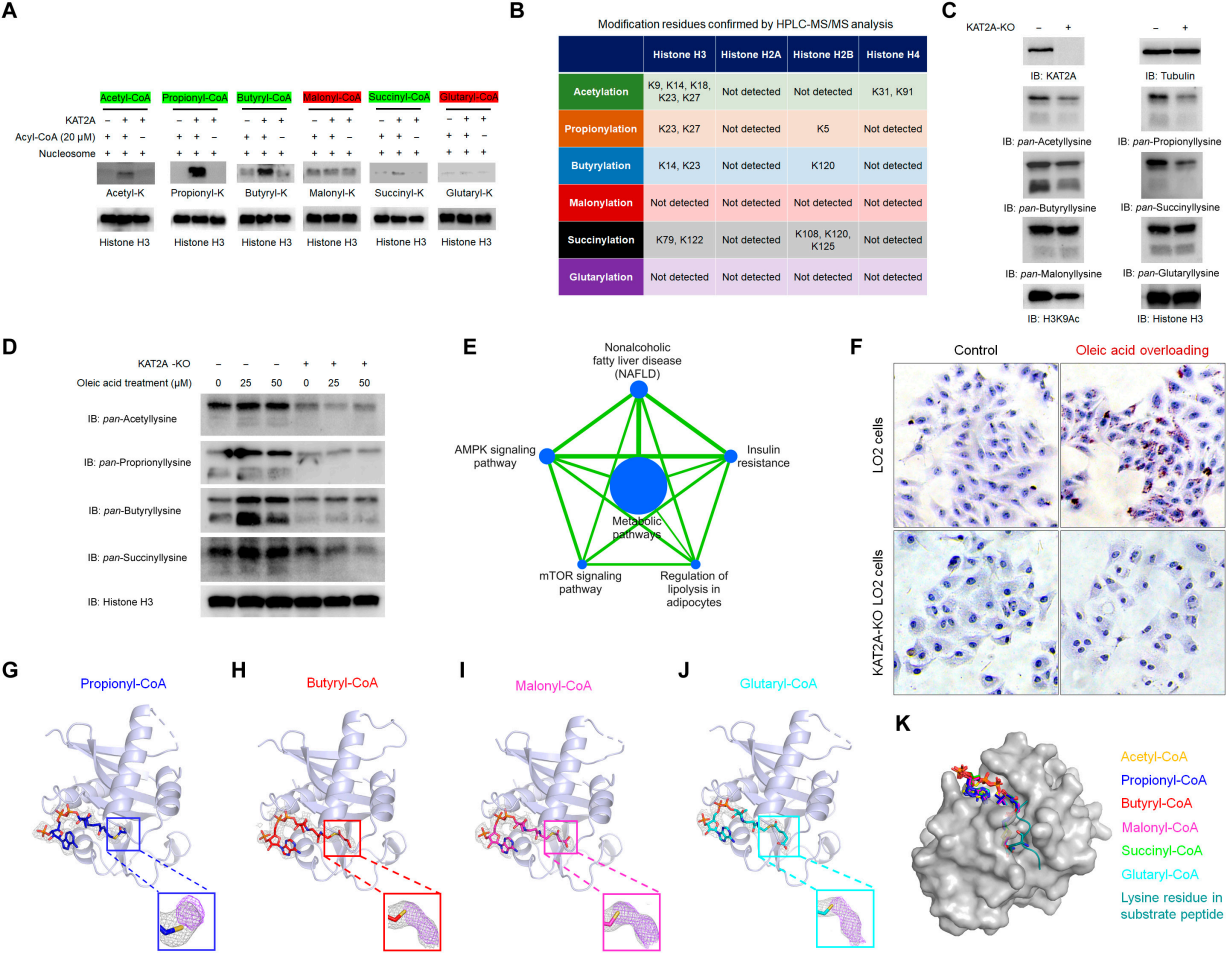
KAT2A is a pluripotent acyltransferase. (A) KAT2A acylates nucleosome using different acyl-CoA cofactors. Immunoblotting analyses were performed with the indicated antibodies. Representative images of triplicate experiments are shown. (B) KAT2A acylates multiple lysine residues of core histones in nucleosome. The modified lysine residues in the KAT2A-mediated in vitro nucleosome acylation assay were identified by performing HPLC-MS/MS analysis. (C) KAT2A regulates histone acylations in cells. The level of histone aceylation, propionylation, butyrylation, malonylation, glutarylation, and succinylation in LO2 cells were analyzed by performing immunoblotting (IB) assays with the indicated antibodies. Data represent 3 independent experiments. (D to F) KAT2A mediates cells responding to oleic acid treatment. Immunoblotting assays were performed with indicated antibodies to show the levels of histone acylation (D). RNA-seq analyses revealing oleic acid treatment-upregulated pathways that were significantly suppressed by the KAT2A depletion (E). Significantly downregulated pathways were identified via Gene Set Enrichment Analysis (*P* < 0.05) and were enriched with downregulated genes. The blue node size reflects the number of significantly suppressed genes in the pathway. Edges represent that more than one gene are shared between the pathways. Oleic acid treatment-induced lipid droplet accumulation is suppressed in cells with KAT2A depletion (F). Oil red O staining was performed to study the lipid droplet accumulation in cells. Representative images of triplicate experiments in immunoblotting and Oil red O staining assays are shown. (G to J) Co-crystal structures of propionyl-CoA (G), butyryl-CoA (H), malonyl-CoA (I), and glutaryl-CoA (J) binding to the catalytic domain of KAT2A, respectively. KAT2A is shown as light blue cartoons. The CoA substrates are shown as sticks in blue (propionyl-CoA), red (butyryl-CoA), magenta (malonyl-CoA), or cyan (glutaryl-CoA). Omit maps were calculated with the (G) propionyl, (H) butyryl, (I) malonyl, or (J) glutaryl moiety removed. 2Fo-Fc maps are contoured at 0.7 to 0.8σ (gray), and simulated annealing omit maps are contoured at 2.5 to 3σ (purple blue). (K) The surface presentation of KAT2A in complex with acetyl-CoA (yellow; PDB ID: 1Z4R), propionyl-CoA (blue; PDB ID: 8H66), butyryl-CoA (red; PDB ID: 8H65), malonyl-CoA (magenta; PDB ID: 8H6C), succinyl-CoA (green; PDB ID: 5TRL), and glutaryl-CoA (cyan; PDB ID: 8H6D) overlaid in the binding pocket. The acyl-CoA cofactors are shown as sticks. The position of substrate peptide is simulated based on the bi-substrate complex structure of *Tetrahymena* GCN5 (PDB ID: 1M1D) shown in deep teal, with the lysine substrate highlighted as sticks.

Nucleosome is the fundamental unit of chromatin in eukaryotic cells [9]. We incubated the purified KAT2A and in vitro assembled nucleosome with acetyl-CoA, propionyl-CoA, butyryl-CoA, malonyl-CoA, succinyl-CoA, and glutaryl-CoA. Immunoblotting analyses revealed that KAT2A can acetylate, propionylate, butyrylate, and succinylate histones (Fig. 1A). We then performed high-performance liquid chromatography-tandem mass spectrometry (HPLC-MS/MS) analyses and found that 18 lysine residues in core histones were modified (Fig. 1B and Supplementary Information). To confirm the versatile histone acyltransferase activities of KAT2A in cells, we depleted KAT2A in LO2 cells. Immunoblotting assays revealed that KAT2A depletion reduced acetylation, propionylation, butyrylation, and succinylation of core histones in the cells, demonstrating that KAT2A has pluripotent histone acyltransferase activities in cells (Fig. 1C). Acyl-CoA molecules are metabolites. KAT2A can mediate the responses of histone acylations (Fig. 1D), transcriptome (Fig. 1E), and lipid droplet accumulation in cells to oleic acid treatment (Fig. 1F), suggesting that KAT2A might translate metabolic states into histone acylation profiles, mediating cellular responses by regulating gene transcription.

## Molecular Basis Underlying KAT2A Selecting Acyl-CoA Substrates

The size of acyl groups was thought as the determinant of acetyltransferases utilizing acyl-CoA [8]. Longer acyl chains have lower efficiency of being utilized by acyltransferases [8]. Charged, branched, or planar acyl-CoA cofactors are also inefficient substrates of HATs [8]. However, our results partially disagreed with these conclusions. Succinyl-CoA is a charged and branched acyl-CoA cofactor, but the succinyltransferase activity of KAT2A is more efficient than its acetyltransferase activity [7]. Although succinyl-CoA is larger than malonyl-CoA, KAT2A can utilize succinyl-CoA but not malonyl-CoA to modify nucleosome (Fig. 1A). These results suggest an alternative mechanism for KAT2A in selecting acyl-CoA substrates.

To understand the molecular basis of KAT2A selecting acyl-CoA cofactors for histone modifications, we co-crystallized and determined the structures of the catalytic domain of KAT2A in complex with propionyl-CoA (2.8-Å resolution; PDB: 8H66), butyryl-CoA (3.0 Å; PDB: 8H65), malonyl-CoA (2.5 Å; PDB: 8H6C), and glutaryl-CoA (3.26 Å; PDB: 8H6D) (Fig. 1G to J and Table S1). Superimposition analyses revealed that all the studied acyl-CoA molecules fit in the same substrate-binding pocket, surrounded by the sheets of β4 and β6 and the helices of α3 and α5 (Fig. 1K), indicating that all the studied acyl-CoA could potentially be utilized by KAT2A to modify histones. However, the malonyltransferase and glutaryltransferase activities of KAT2A were not detected.

**Fig. 2.**
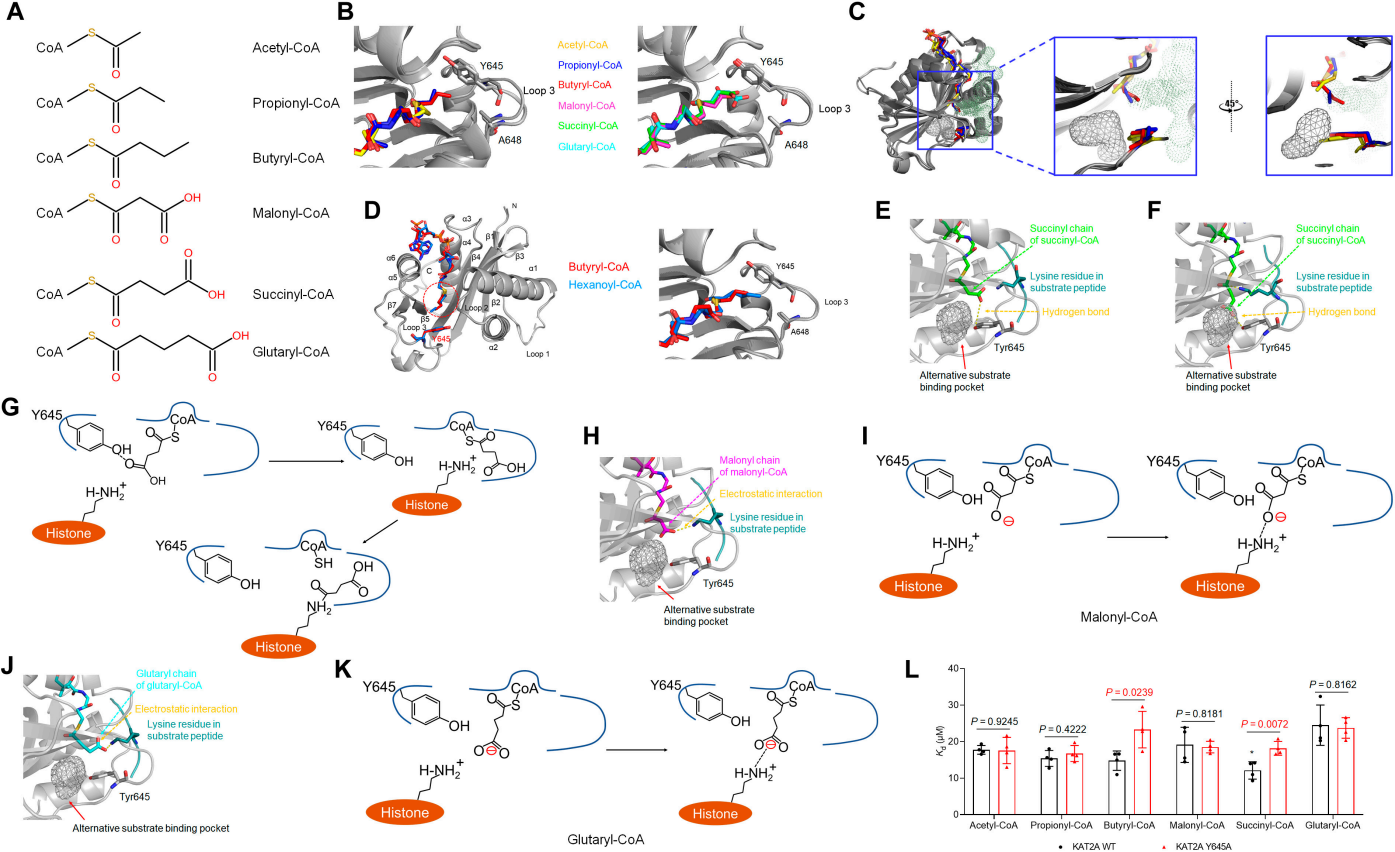
Molecular basis of KAT2A selecting acyl-CoA substrates. (A) Illustration of chemical structures of acetyl-CoA, propionyl-CoA, butyryl-CoA, malonyl-CoA, succinyl-CoA, and glutaryl-CoA. (B) Superimposition of interactions between acyl-CoA cofactors and Loop 3 of KAT2A, with type I acyl-CoA in the left and type II acyl-CoA in the right. The catalytic domain of KAT2A is shown in gray. Tyr645, Ala648, and the acyl-CoA cofactors are shown as sticks. Structures of KAT2A in complex with type I acyl-CoA: KAT2A–acetyl-CoA (PDB ID: 1Z4R), KAT2A–propionyl-CoA (PDB ID: 8H66), and KAT2A–butyryl-CoA (PDB ID: 8H65). Structures of KAT2A in complex with type II acyl-CoA: KAT2A–malonyl-CoA (PDB ID: 8H6C), KAT2A–succinyl-CoA (PDB ID: 5TRL), and KAT2A–glutaryl-CoA (PDB ID: 8H6D). (C) Location of the alternative pocket of the substrate binding sites of KAT2A with respect to type I acyl-CoA molecules. The catalytic domain of KAT2A is shown in gray. Acyl-CoA cofactors are shown as sticks. The alternative pocket is shown as a gray mesh, and the lysine residue in substrate peptide that is positioned based on histone H3 (PDB ID: 1M1D) is shown as green dots. The 2 enlarged panels, viewed with a 45° rotation, display the alternative pocket of the substrate binding sites of KAT2A. Structures of KAT2A in complex with type I acyl-CoA: KAT2A–acetyl-CoA (PDB ID: 1Z4R), KAT2A–propionyl-CoA (PDB ID: 8H66), and KAT2A–butyryl-CoA (PDB ID: 8H65). (D) Docking analysis of hexanoyl-CoA binding to the catalytic domain of KAT2A. The left is the superimposition of the KAT2A–butyryl-CoA complex and KAT2A–hexanoyl-CoA complex structures. The latter is simulated on the basis of the former. KAT2A is shown in gray. Butyryl-CoA is shown as red sticks, and hexanoyl-CoA is shown as marine sticks. The enlarged panel displays the interaction between acyl-CoA cofactors and Loop 3 of KAT2A. Tyr645 and Ala648 are shown as sticks. The different carbon atoms between the 2 CoA chains are highlighted with a red dashed circle. (E to K) Structural models and catalytic reaction illustration of succinyl-CoA (PDB ID: 5TRL) (E to G), malonyl-CoA (PDB ID: 8H6C) (H and I), and glutaryl-CoA (PDB ID: 8H6D) (J and K) flipping or not flipping into the alternative pocket of the substrate binding sites of KAT2A. Models (E), (F), (H), and (J) show KAT2A as gray cartoons, succinyl-CoA as green sticks, malonyl-CoA as magenta sticks, glutaryl-CoA as cyan sticks, lysine residue in substrate peptide as deep teal sticks, alternative pocket as a gray mesh, and hydrogen bonds as yellow dotted lines. For the reaction illustrations (G, I, and K), KAT2A is shown as blue lines; Tyr645 of KAT2A, succinyl-CoA, and the lysine residue of substrate peptide are shown as chemical structures. (L) The binding affinities of KAT2A to different acyl-CoA cofactors. Thermofluor shift assays were performed to study the dissociation constant (*K*_d_) of wild-type (WT) KAT2A and KAT2A Y645A mutant binding to acetyl-CoA, propionyl-CoA, butyryl-CoA, malonyl-CoA, succinyl-CoA, and glutaryl-CoA. *n* = 4 biologically independent samples; two-sided *t* test were conducted to calculate the *P* value, and the data are presented as the means ± SD.

The studied acyl-CoA molecules can be categorized into 2 types. Acetyl-CoA, propionyl-CoA, and butyryl-CoA are type I CoA molecules without a carboxyl group at the end of the acyl chains (Fig. 2A). As the acyl chain gets longer, the terminus extends to reach deeper into the binding pocket toward Loop 3 (Fig. 2B), which might occupy the space for the incoming histone lysine residue [10]. KAT2A has an alternative pocket for substrate binding [7]. The propionyl and butyryl chain could flip into the alternative pocket (Fig. 2C), orienting the reactive carbonyl group of their acyl chain to receive proton transferred from the ε-amino group of the lysine substrate, allowing the covalent modification to occur. Thus, propionyl-CoA and butyryl-CoA can be utilized by KAT2A for histone modifications (Fig. 1A). By replacing the acyl-CoA with a hexanoyl-CoA model, we simulated the accommodation of hexanoyl-CoA which fits well in the KAT2A catalytic pocket (Fig. 2D), suggesting that KAT2A could potentially utilize acyl-CoAs with larger acyl chain than that of butyryl-CoA.

Malonyl-CoA, succinyl-CoA, and glutaryl-CoA are type II CoA molecules with a carboxyl group at the end of the acyl chains (Fig. 2A). The negatively charged terminal carboxyl group interacts with the positively charged ε-amino of the lysine substrate in the pocket. This electrostatic interaction could theoretically disrupt the transfer of the acyl group from the acyl-CoA substrates to the lysine substrate. This could explain the lower efficiency of KAT2A utilizing the charged acyl-CoA cofactors for histone acylations, despite the size of malonyl-CoA being similar to butyryl-CoA (Fig. 2A).

Succinyl-CoA is an exception. Given its length, the succinyl group precisely positions its terminal carboxyl group to form a hydrogen bond with the Tyr645 residue of KAT2A [7]. This interaction could redistribute the electron density of the terminal carboxyl group toward the Tyr645 residue, which prevent the nonproductive electrostatic interactions between the succinyl group and the positively charged ε-amino group of the lysine substrate (Fig. 2E), allowing the succinyl chain, similar to the neutral acyl chain, being flipped into the alternative pocket for the succinylation process (Fig. 2F and G). On the contrary, the acyl chain of malonyl-CoA and glutaryl-CoA is either too short or too long, and turned aside, to form hydrogen bonds with the Tyr645 residue (Fig. 2H to K), so that the electrostatic interaction between the malonyl/glutaryl moiety and lysine residue blocked malonylation/glutarylation (Fig. 2I and K). Consistently, the mutation of Tyr645 only reduced KAT2A binding to succinyl-CoA but showed no influence on the binding affinity of KAT2A to malonyl-CoA and glutaryl-CoA (Fig. 2L). The binding affinity of KAT2A to butyryl-CoA was unexpectedly affected by the Y645A mutation (Fig. 2L), indicating an additional interaction between the butyryl chain and the Tyr645 residue in KAT2A that was not noted previously. The interface analysis of the current structures showed a buried surface area of ~10 Å^2^ [2] between Tyr645 and the butyryl chain, which is comparable to that between Tyr645 and the succinyl chain of ~7 Å^2^ [2]. Instead of the hydroxyl group that forms a hydrogen bond with the succinyl chain, it is the benzene ring of Tyr645 that is involved in the interaction with the butyryl chain. The exact properties and biochemical functions of this interaction will be further investigated.

In summary, KAT2A can selectively utilize acetyl-CoA, propionyl-CoA, butyryl-CoA, and succinyl-CoA to directly modify histones in nucleosome. The molecular basis of KAT2A selecting acyl-CoA cofactors is cooperatively influenced by the length and electrostatic characteristics of acyl chains and the alternative substrate-binding pocket of KAT2A.

## Discussion

Histone modification landscapes determine the transcriptomes and biological processes that usually match the physiological states of cells. The fatty acid-overloading nutrient-induced abnormal distribution of acyl-CoA might be sensed by KAT2A. This pluripotent acyltransferase manipulates the corresponding histone acylation landscape that controls cell response to particular stresses, such as lipid droplet accumulation. Depletion of KAT2A might disconnect histone acylation from the abnormalities of acyl-CoA in the cell, resulting in an inaccurate response to the fatty acid-overloading nutrient.

While the molecular basis of a certain acyl-CoA selection was illustrated in this study, how KAT2A selects lysine residues for histone modifications is a parallel mechanism remaining elusive, as KAT2A selects both acyl-CoAs and lysine residues to precisely paint histone acylation landscapes. The nucleus is an alternative subcellular pool of acyl-CoA molecules [11]. The pluripotent acyltransferase activity could be a feature of HATs, so that it is worthy asking whether the model of “one HAT, one histone hallmark, one function” could mirror the functions of HATs in cells. It will be challenging to answer these questions, yet it would pave a brilliant avenue to advance the field of histone modification.

## Materials and Methods

The materials and methods descriptions are captured in Supplementary Information.

## Data Availability

The data that support the findings of this study are included in here and the Supplementary Materials. The RNA-seq data have been deposited in the Genome Sequence Archive for Human (GSA-Human) with the accession number HRA003976 (https://bigd.big.ac.cn/gsa-human/browse/HRA003976). The crystal structural coordinates were deposited at the RCSB Protein Data Bank (PDB ID 8H66, 8H65, 8H6C, and 8H6D for the propionyl-CoA, butyryl-CoA, malonyl-CoA, and glutaryl-CoA structures, respectively.

## Supplementary Materials

Supplementary 1 Materials and Methods

Supplementary 2 Mass Spectrums

Supplementary 3 Table S1. Crystallographic statistics of co-crystal structures.
