# Supplementary material for: Molecular Basis of KAT2A Selecting Acyl-CoA Cofactors for Histone Modifications
Source: Research (Wash D C). 2023 Apr 4;6:0109. doi: 10.34133/research.0109 (PMC10076270; doi:10.34133/research.0109)
Supplement: Supplementary 1 — Materials and Methods [file research.0109.f1.docx]

**Molecular basis of KAT2A selecting acyl-CoA cofactors for histone modifications**

Sha Li^1#^, Nan Li^1#^, Jie He^2#^, Runxin Zhou^1^, Zhimin Lu^3^, Yizhi Jane Tao^4^, Yusong R. Guo^1,5^*, Yugang Wang^1,5^*

^1^Department of Biochemistry and Molecular Biology, School of Basic Medicine, Tongji Medical College, Huazhong University of Science and Technology, Wuhan, Hubei, 430030, China. ^2^Department of Neurosurgery, Union Hospital, Tongji Medical College, Huazhong University of Science and Technology, Wuhan, 430022, China. ^3^Zhejiang University School of Medicine, Hangzhou, Zhejiang, 310029, China. ^4^Department of Bioscience, Rice University, 77005, United States. ^5^Cell Architecture Research Center, Huazhong University of Science and Technology, Wuhan, Hubei, 430030, China.

# These authors contributed equally to this work.

*Correspondence author. E-mail: yrguo@hust.edu.cn (Y.R.G); yugangw@hust.edu.cn (Y.W.)

**Materials and Methods**

**Materials**

Primary antibodies for immunoblotting: β-actin (mouse, Cat#AC004, Abclonal, Wuhan, China), Acetyl-lysine (rabbit, Cat#9441S, Cell Signaling Technology, MA, USA), propionyl-lysine (rabbit, Cat#PTM-201, PTM Biolabs, Hangzhou, China), butyryl-lysine (rabbit, Cat#PTM-301, PTM Biolabs, Hangzhou, China), malonyl-lysine (rabbit, Cat#PTM-901, PTM Biolabs, Hangzhou, China), succinyl-lysine (rabbit, Cat#PTM-401, PTM Biolabs, Hangzhou, China), glutaryl-lysine (rabbit, Cat#PTM-1151, PTM Biolabs, Hangzhou, China), histone H3 (rabbit, Cat#4499S, Cell Signaling Technology, MA, USA), KAT2A (rabbit, Cat#3305S, Cell Signaling Technology, MA, USA), H3K9-acetylation (rabbit, Cat#9649S, Cell Signaling Technology, MA, USA). Recombinant KAT2A (Cat#31591, Active Motif, CA, USA). *In vitro* assembled nucleosome (Cat#81070, Active Motif, CA, USA). The sgRNA sequence for CRISPR/Cas9-mediated KAT2A knockout is CACCGATTACACTTACAGGTTTCAT.

**Cell culture**

Human hepatocyte LO2 cells were kindly provided by Dr. Weidong Xie at Tsinghua University. The cells were cultured in Dulbecco’s modified Eagle’s medium (Cat#SH30285.01, Cytiva Life Sciences, MA, USA) supplemented with 10% fetal bovine serum (Cat#086-150, WISENTINC, Quebec, Canada) and 1% penicillin and streptomycin (Cat#BL505A, biosharp, Beijing, China). The cultured cells were kept at 37℃ in the cell culture incubator (CLM-170B-8-NF, ESCO, Singapore) with 5% CO_2_.

***In vitro* KAT2A acyltransferase activity assays**

To determine the acyltransferase activities of KAT2A, we incubated purified full-length active KAT2A and *in vitro* assembled nucleosome in the presence of HAT buffer (50 mM Tris-HCl, pH 8.0, 50 mM KCl, 0.1 mM EDTA, 1 mM dithiothreitol, 1 mM PMSF, 10 mM sodium butyrate) and 2 μM acyl-CoA (acetyl-CoA, propionyl-CoA, butyryl-CoA, malonyl-CoA, succinyl-CoA, and glutaryl-CoA) at 37 °C for 10 min. Histone acylation was determined by using immunoblotting analysis. The modified lysine residues on histones were identified using HPLC-MS/MS analyses.

**RNA-sequencing and analysis**

Total RNA was extracted from cells using the RNA simple total RNA kit (#DP419, TIANGEN, Beijing, China). Illumina compatible libraries were prepared using the NEBNext® Ultra™ RNA Library Prep Kit for Illumina®. In brief, using fragmented mRNA as a template and random oligonucleotides as primers, the first strand of cDNA was synthesized in the M-MuLV reverse transcriptase system. In the DNA polymerase I system, dNTPs were used as the primer. The raw material was used to synthesize the second strand of cDNA. The purified double-stranded cDNA underwent end-repair, A-tailing and ligation of sequencing adapters. The 250-300bp cDNA was screened with AMPure XP beads, PCR amplification was performed, and the PCR products were purified again with AMPure XP beads to obtain the library. After the library was constructed. Qubit 2.0 Fluorometer was used for preliminary quantification, dilute the library to 1.5 ng/ul, and then used Agilent 2100 bioanalyzer to detect the insert size of the library. After the insert size meets the expectation, real-time RT-PCR was used to measure the effective concentration of the library. The hallmark gene sets were used in the GSEA analysis were downloaded from MSigDB (https://www.gsea-msigdb.org/gsea/msigdb/). GSEA results were visualized using the Enrichment Map v3.3.3 plugin of Cytoscape v3.8.2.

**Thermofluor Shift Assay**

SYPRO Orange Protein Gel Stain (Cat#S5692, Sigma-Aldrich, St. Louis, USA) were diluted 1:2500 into the assay buffer (150 mM NaCl, 200 mM Tris-Cl pH 7.5, 10 mM MgCl_2_, 20 mM β-Mercaptoethanol). ALDH1A1 (WT/Mutant) or IDH1 (WT/Mutant) was added with the final concentration of 0.2 μM, respectively. The 50 μl mixture were loaded into PCR 8-Strip Tubes (Cat#CP0101, GSBIO, Wuxi, China). Melting curves were obtained applying a temperature gradient from 25 to 99 °C and a heating rate of 0.5 °C/s. The fluorescence intensity was measured by a CFX Connect™ Real-Time PCR System (Cat#1855201, Bio-Rad, USA).

**Oil Red O Staining**

The Oil red O staining kit (Cat#: G1262, Solarbio, Beijing) was used to study the lipid droplet accumulation in cells. The experiments were performed according to the manufacturer instructions.

**Crystallization, data collection and structure determination**

The catalytic domain of KAT2A was purified and co-crystallized with acyl-CoA substrate as previously described^1^. In brief, 10 mM propionyl-CoA, butyryl-CoA, malonyl-CoA, succinyl-CoA, and glutaryl-CoA was added to KAT2A solution at the concentration of 4 mg/ml. Crystallization mother liquor contained 1.4-1.6M Ammonium sulfate, 0.1M Sodium acetate pH 4.6 for propionyl-CoA, butyryl-CoA and malonyl-CoA complex, and 1.3M Ammonium sulfate, 0.4M Lithium chloride for glutaryl-CoA complex. X-ray diffraction data were collected at 77 K by the Life Sciences Collaborative Access Team at the Advanced Photon Source. Data for the glutaryl-CoA complex were collected at a wavelength of 0.97872 Å at Beamline 21-ID-F; data for the propionyl-CoA, butyryl-CoA and malonyl-CoA complex were collected at a wavelength of 0.97857 Å at Beamline 21-ID-G.

Diffraction images were processed using HKL-2000 software. For structure determination, the apo human KAT2A structure (PDB ID: 5TRM) was used for molecular replacement. Molecular replacement was calculated using the Phaser software program in the PHENIX software suite^2^. The structure models were then manually adjusted using the Coot software program^3^ and refined using PHENIX. Structure figures were prepared using the PyMOL molecular graphics system (Version 1.8.6 Schrödinger, LLC). The coordinates have been deposited at the RCSB Protein Data Bank under accession numbers 8H66, 8H65, 8H6C and 8H6D for the propionyl-CoA, butyryl-CoA, malonyl-CoA, succinyl-CoA, and glutaryl-CoA complex structures, respectively.

**Immunoblotting**

Proteins were extracted from cultured cell using 0.5% SDS lysis buffer containing protease, deacetylase and phosphatase inhibitor cocktail. Immunoblotting analyses with indicated antibodies were performed as described previously ^1^.

**Mass spectrometric analysis**

The peptide samples were loaded onto a C18 column (15 cm lengthⅹ0.075 mm ID, 2µm particle size, Thermo Scientific company, Catalog # 164940) connected to a Dionex Ultimate 3000 RSLC nano system (Thermo Scientific Company). Peptides were separated and eluted with a gradient of 8% to 98% HPLC buffer B (80% ACN in 0.1% FA, v/v) in buffer A (0.1% FA in H_2_O, v/v) at a flow rate of 300 nl min^-1^ over 65 minutes. In the comparison between synthesized peptides and their counterparts in cells, the peptides were separated and eluted with a gradient of 1% to 98% HPLC buffer B (80% ACN in 0.1% FA, v/v) in buffer A (0.1% FA in H_2_O, v/v) at a flow rate of 800 nl min^-1^ over 30 minutes. The eluted peptides were then ionized and analyzed by an Orbitrap Fusion Lumos mass spectrometer (Thermo Scientific). Full mass spectrometry was acquired in the Orbitrap mass analyzer over the range *m/z 350* to1,800 with a resolution of 60000 at *m/z* 200. The 20 most intense ions with charge 2-7 were fragmented with normalized collision energy of 28 and tandem mass spectra were acquired with a mass resolution of 15000 at *m/z* 200.

**Supplementary references:**

1 Wang, Y. *et al.* KAT2A coupled with the alpha-KGDH complex acts as a histone H3 succinyltransferase. *Nature* **552**, 273-277, doi:10.1038/nature25003 (2017).

2 Adams, P. D. *et al.* PHENIX: a comprehensive Python-based system for macromolecular structure solution. *Acta Crystallogr D Biol Crystallogr* **66**, 213-221, doi:10.1107/S0907444909052925 (2010).

3 Emsley, P., Lohkamp, B., Scott, W. G. & Cowtan, K. Features and development of Coot. *Acta Crystallogr D Biol Crystallogr* **66**, 486-501, doi:10.1107/S0907444910007493 (2010).
