# Supplementary material for: Molecular Basis of KAT2A Selecting Acyl-CoA Cofactors for Histone Modifications
Source: Research (Wash D C). 2023 Apr 4;6:0109. doi: 10.34133/research.0109 (PMC10076270; doi:10.34133/research.0109)

Acetylation

DNIQGITK(+42.01)PAIR Histone H4 Lys-31

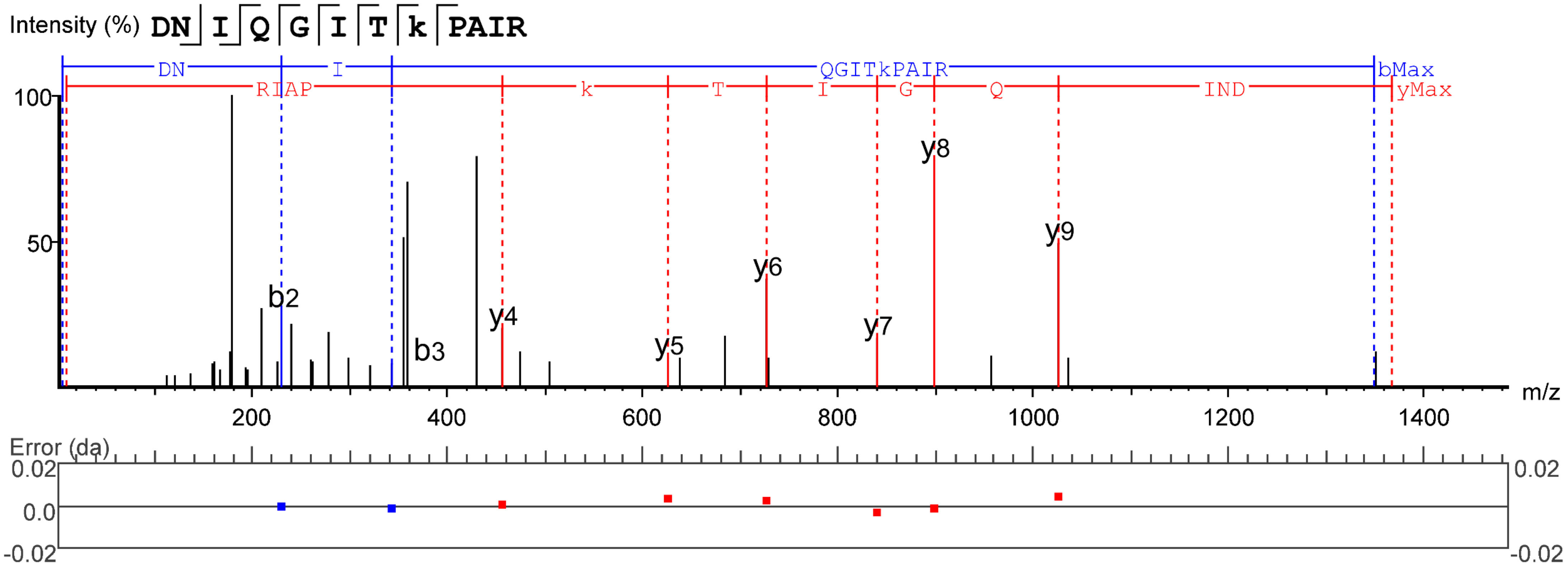

Acetylation

K(+42.01)SAPATGGVK(+42.01)KPHR   Histone H3 Lys-27, Lys-36

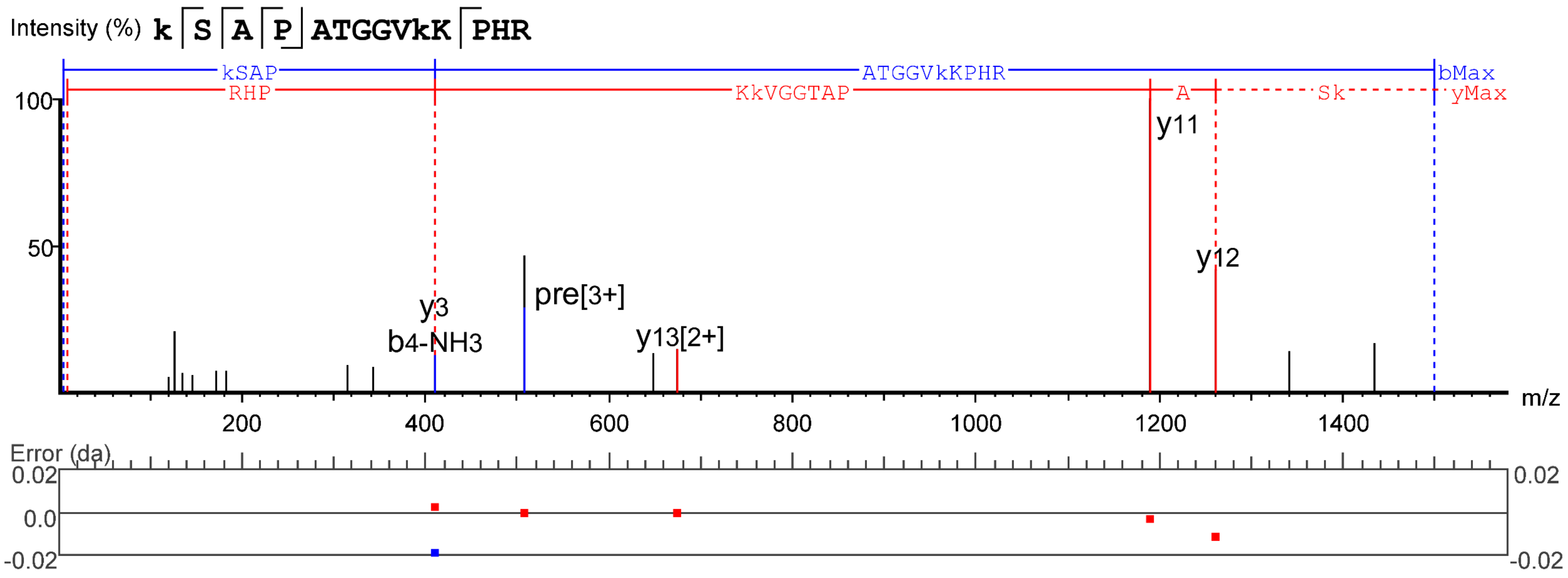

Acetylation

TVTAMDVVYALK(+42.01)R

Histone H4 Lys-91

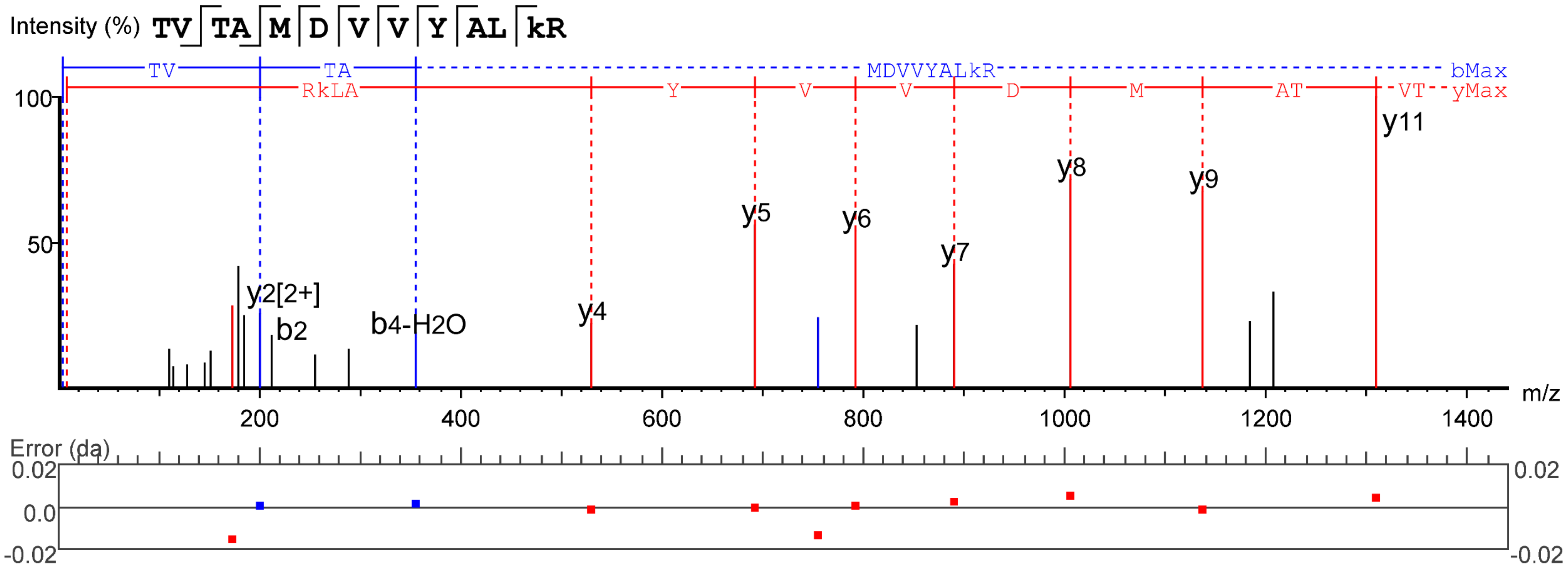

Acetylation

K(+42.01)QLATK(+42.01)AARK   Histone H3 Lys-18, Lys-23

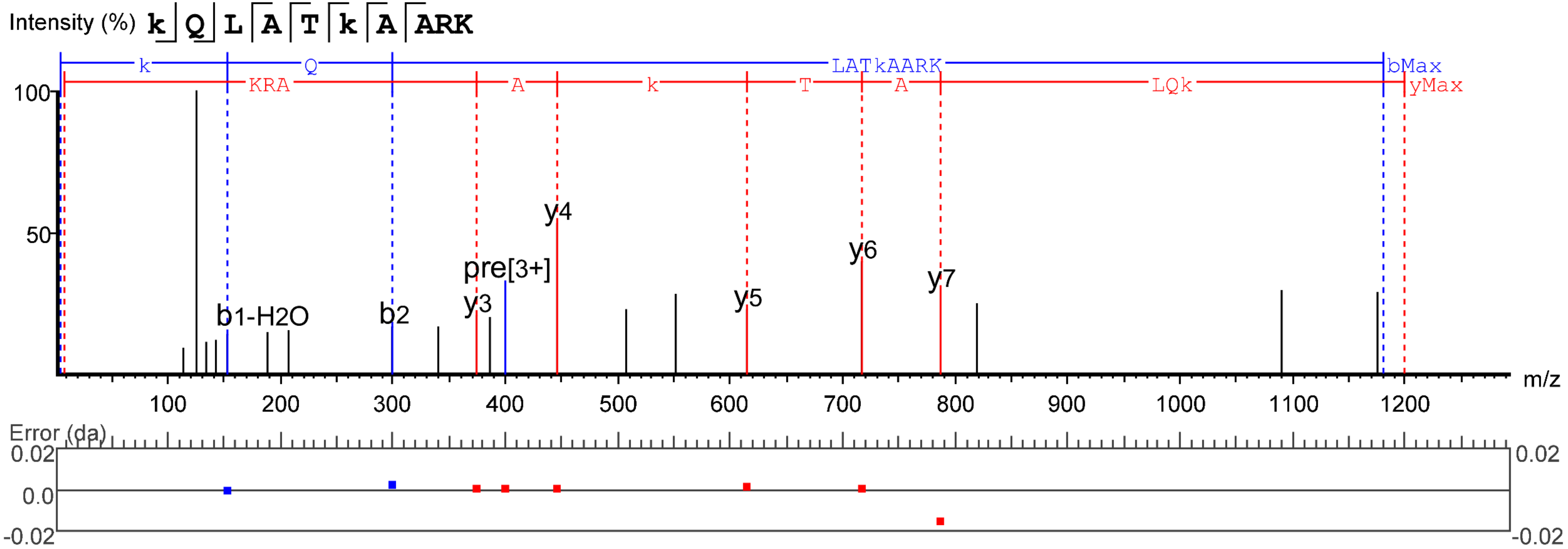

Acetylation

K(+42.01)STGGK(+42.01)APR

Histone H3 Lys-9, Lys-14

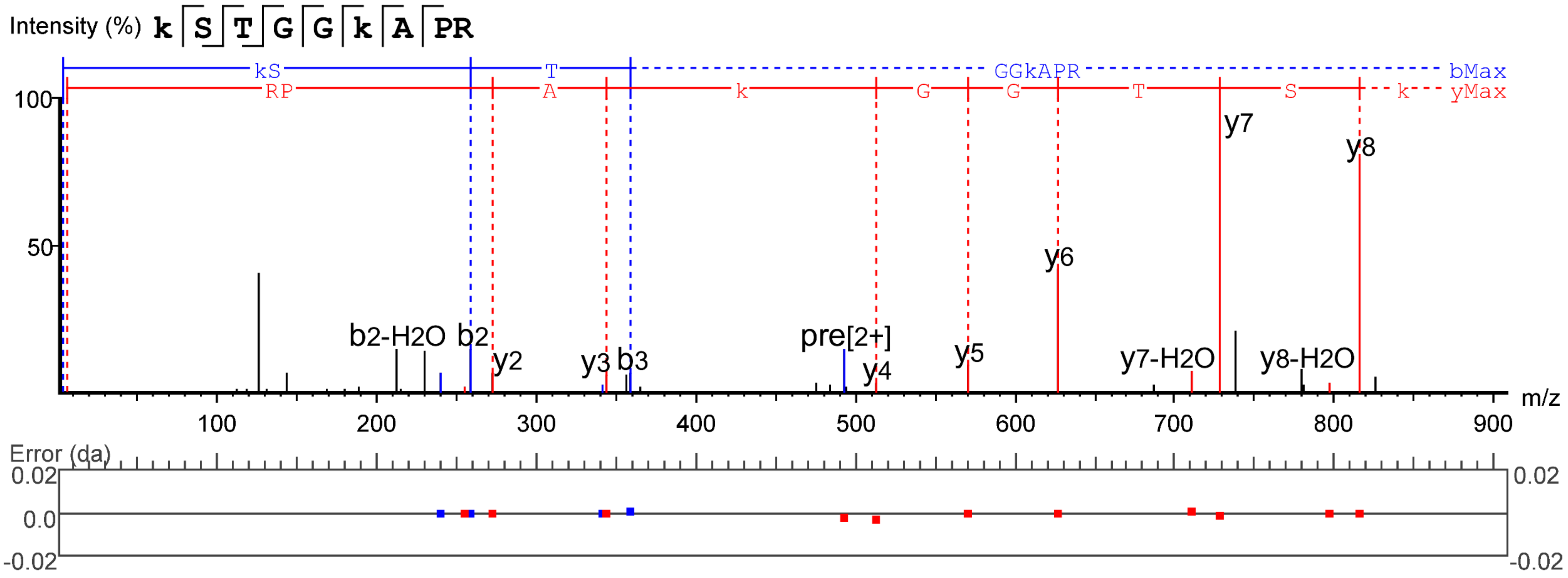

Acetylation

QLATK(+42.01)AAR   Histone H3 Lys-23

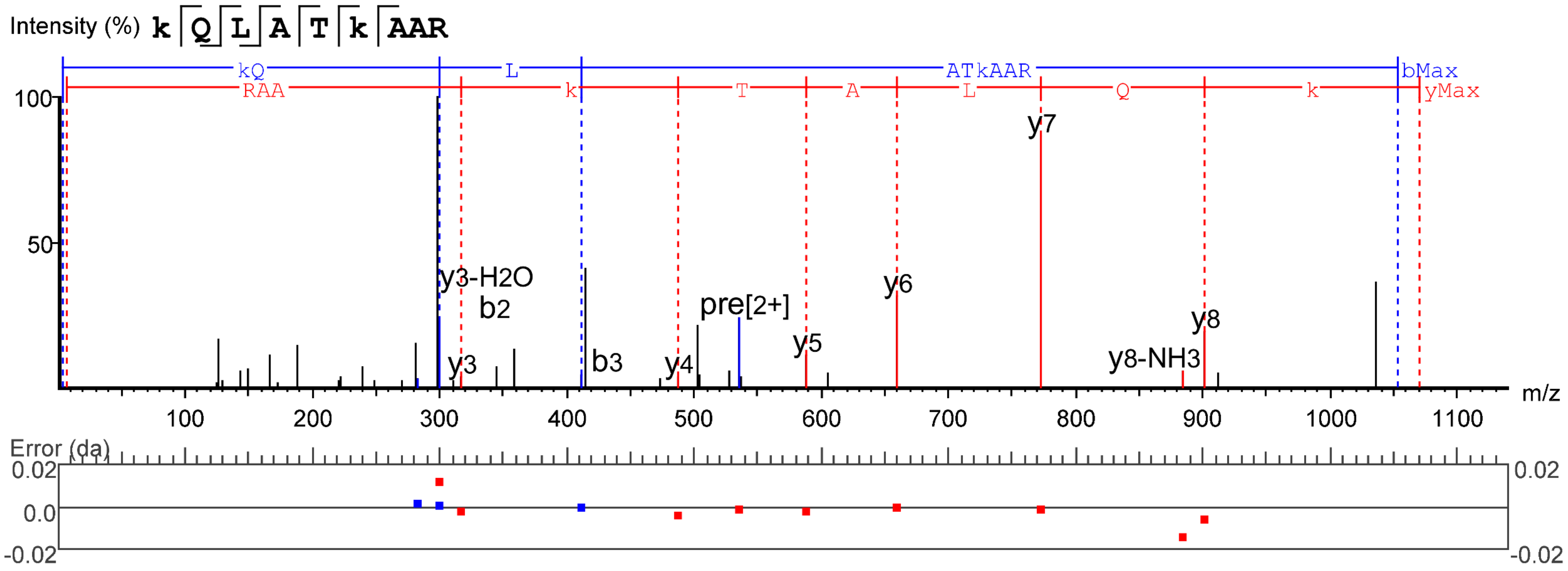

Acetylation

K(+42.01)QLATK(+42.01)AAR Histone H3 Lys-18, Lys-23

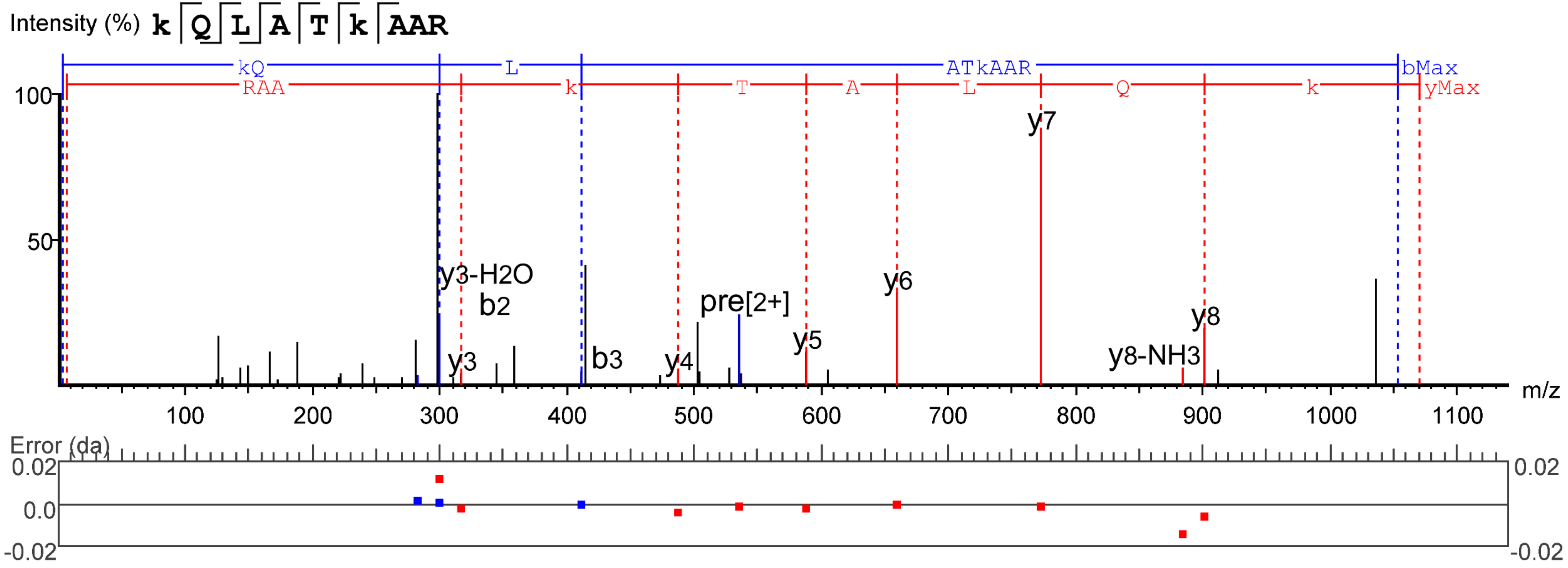

Propionylation

PEPAK(+56.03)SAPAPK

Histone H2B Lys-5

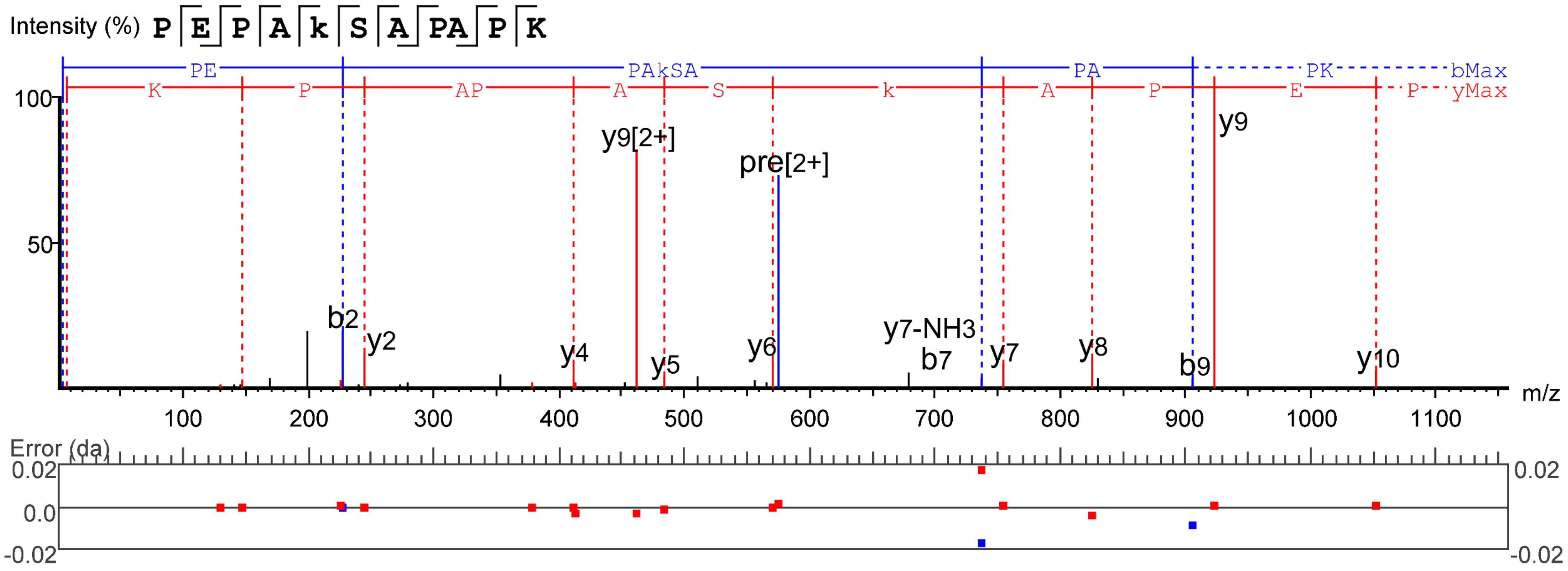

Propionylation

K(+56.03)SAPATGGVK Histone H3 Lys-27

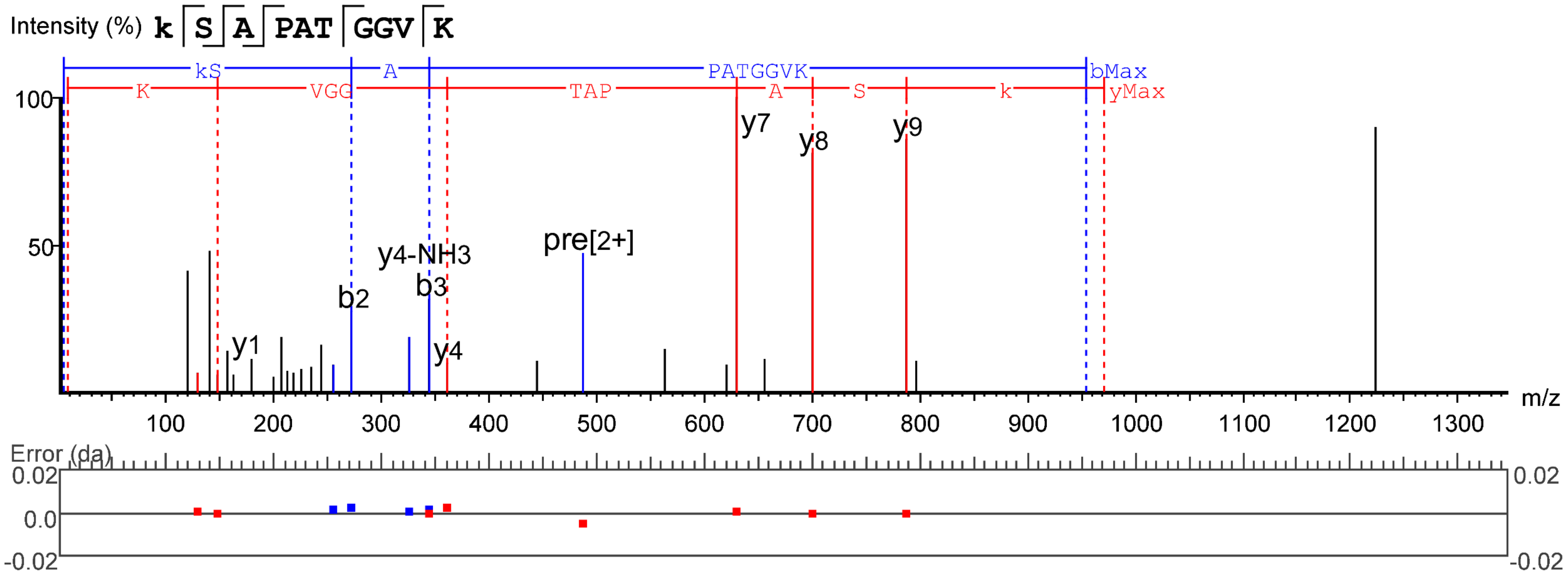

Propionylation

KQLATK(+56.03)AAR

Histone H3 Lys-23

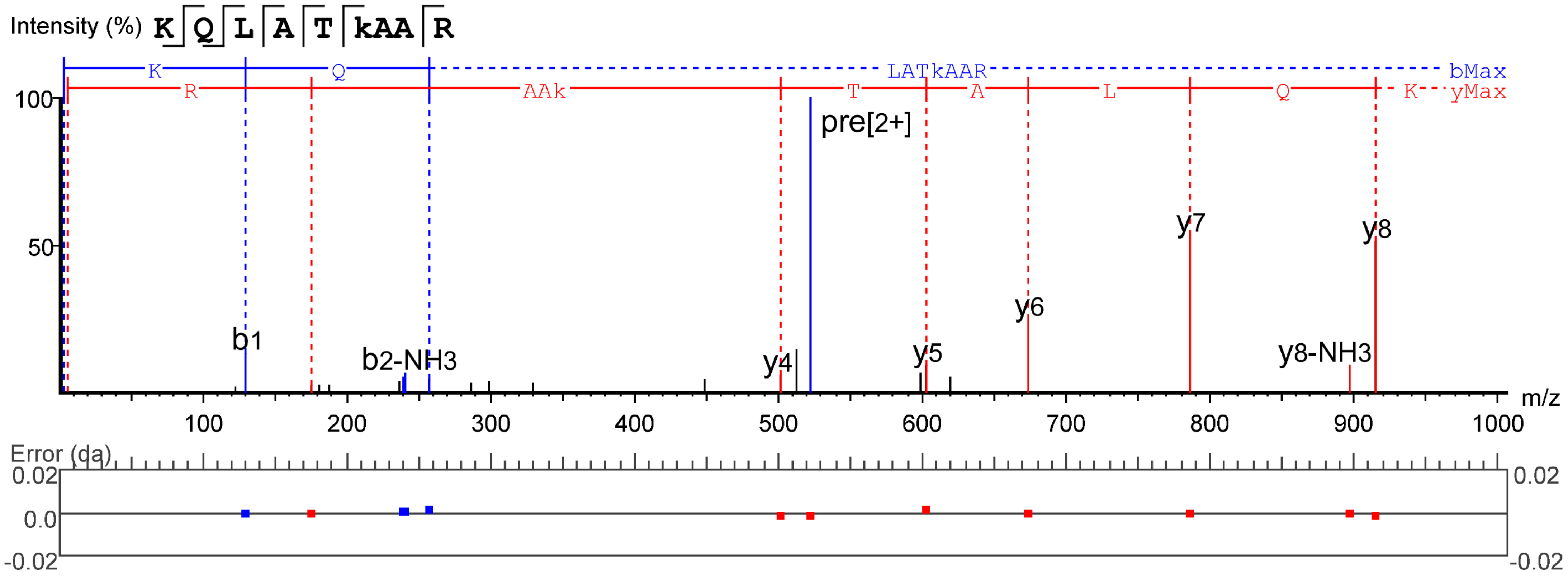

Succinylation

IAQDFK(+100.02)TDLR

Histone H3 Lys-79

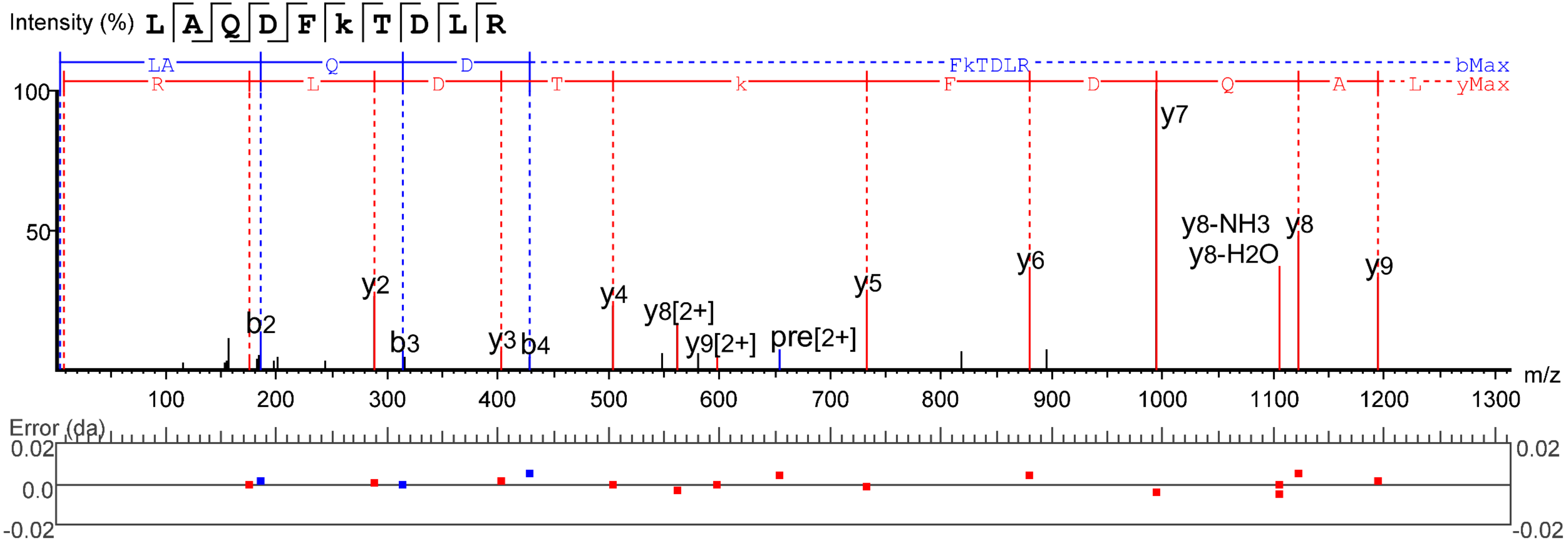

Succinylation

GTKAVTK(+100.02)YTSSK Histone H2B Lys-120

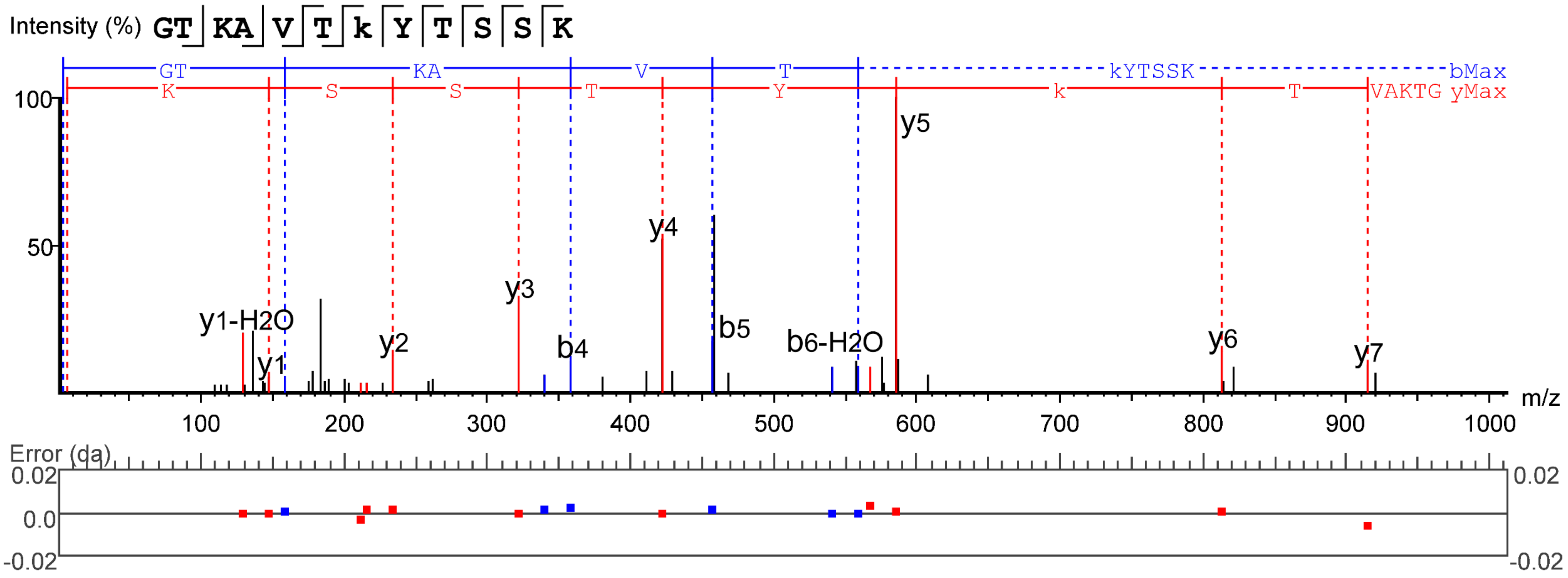

Succinylation

LAK(+100.02)HAVSE Histone H2B Lys-108

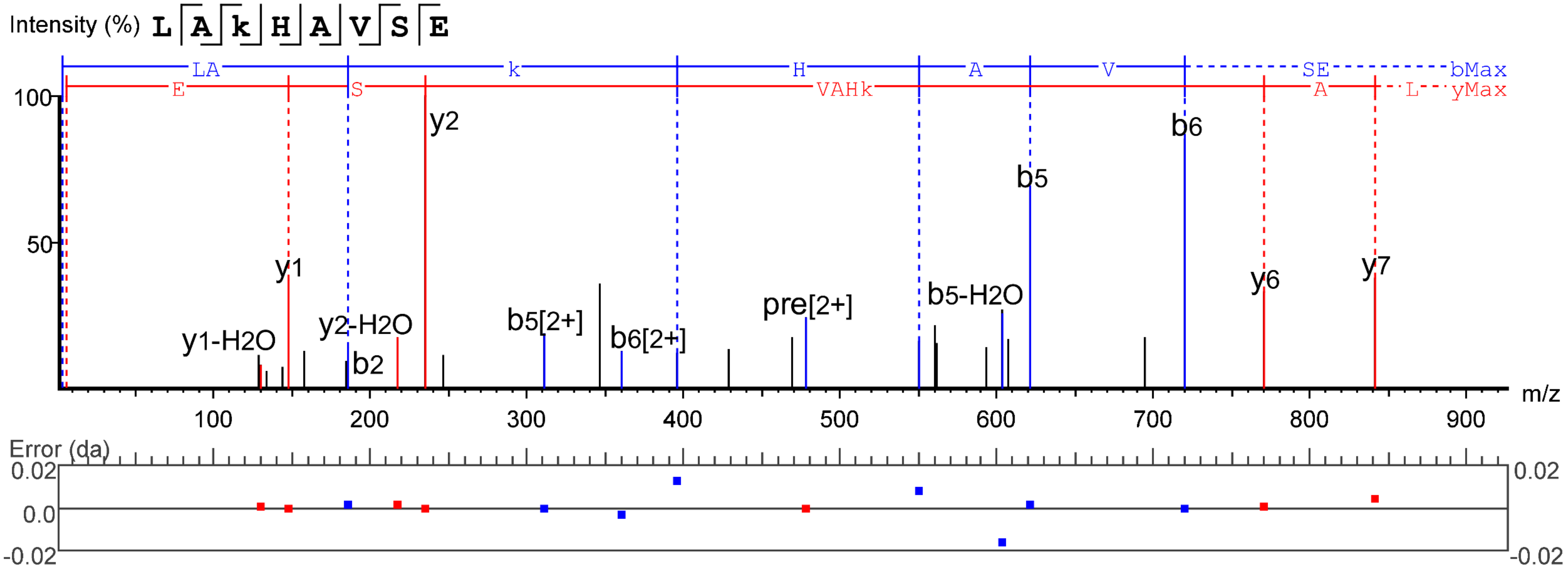

Succinylation

AVTKYTSSK(+100.02)

Histone H2B Lys-125

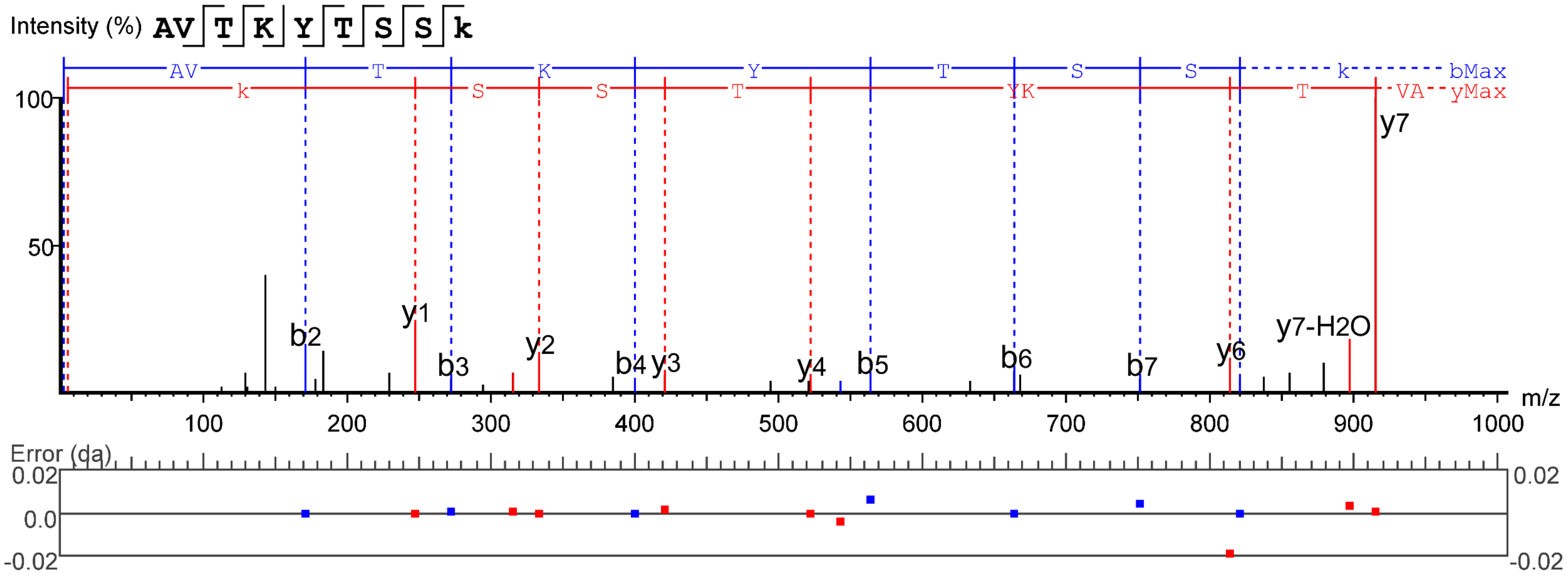

Succinylation

AVTK(+100.02)YTSSK

Histone H2B Lys-120

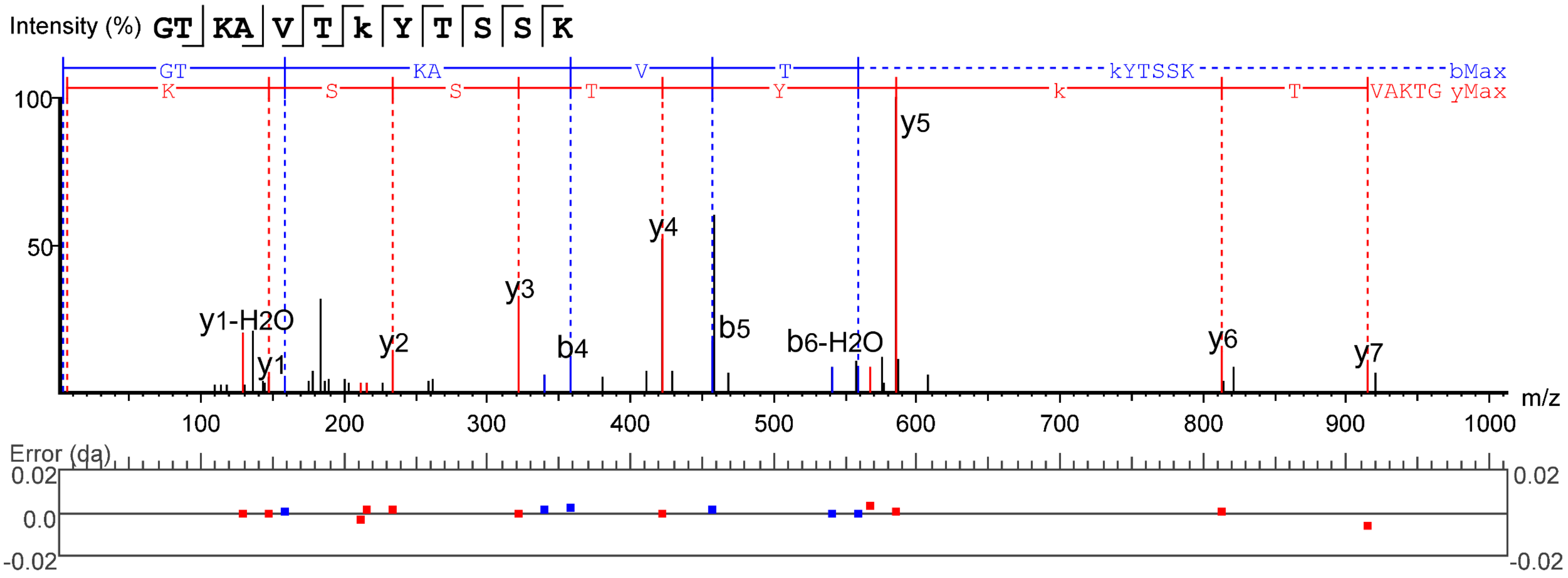

Succinylation

LLLPGELAK(+100.02)HAVSE Histone H2B Lys-108

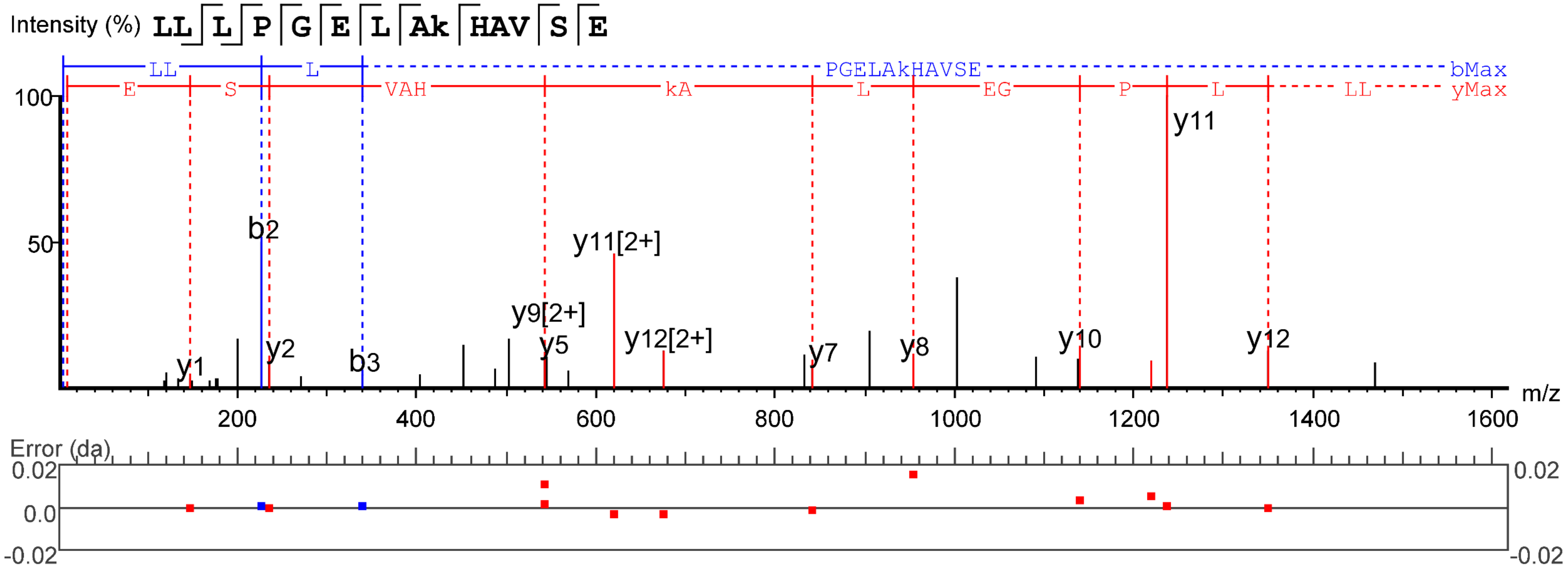

Succinylation

VTIMPK(+100.02)DIQLAR

Histone H3 Lys-122

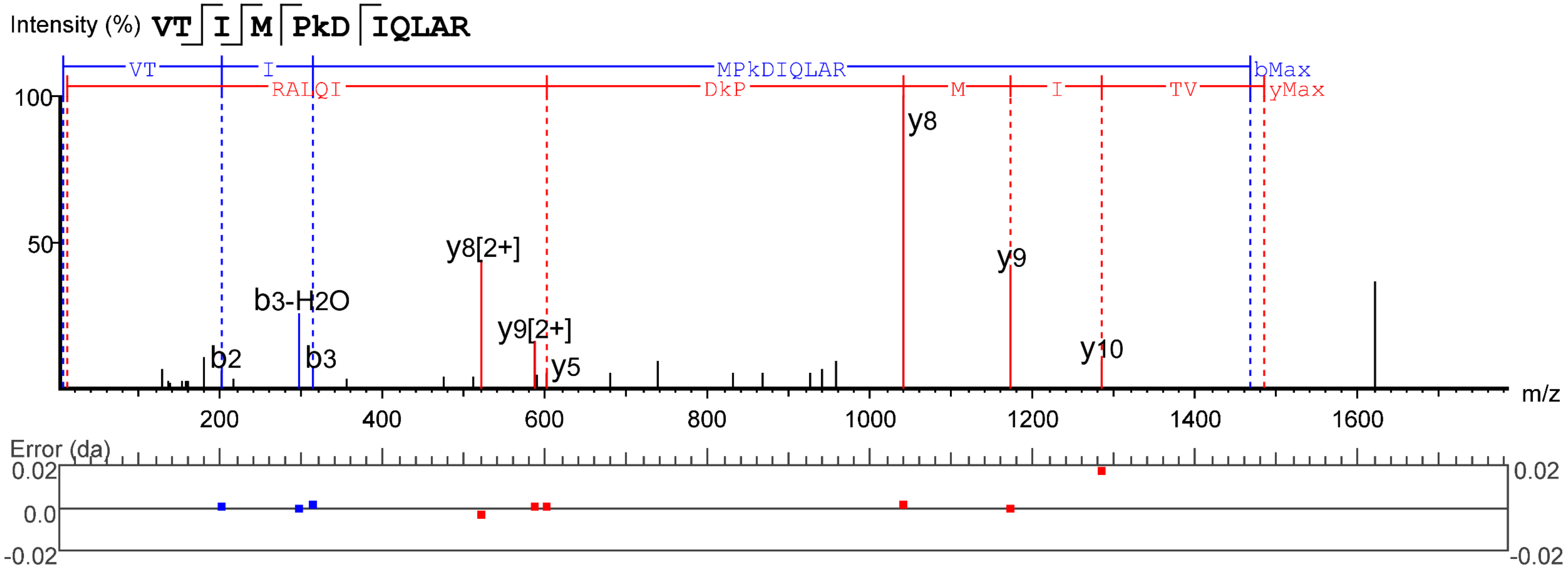

Butyrylation

KSTGGK(+70.04)APRKQLATK Histone H3 Lys-14

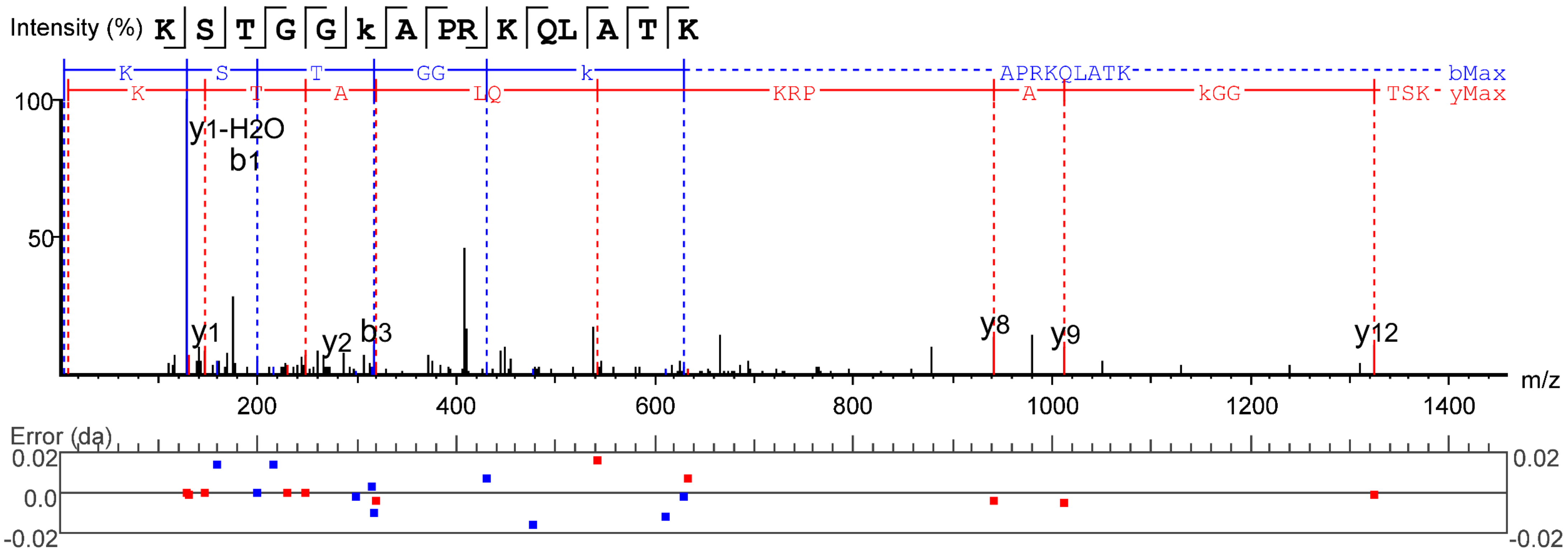

Butyrylation

KSTGGK(+70.04)APRKQLATK

Histone H3 Lys-14

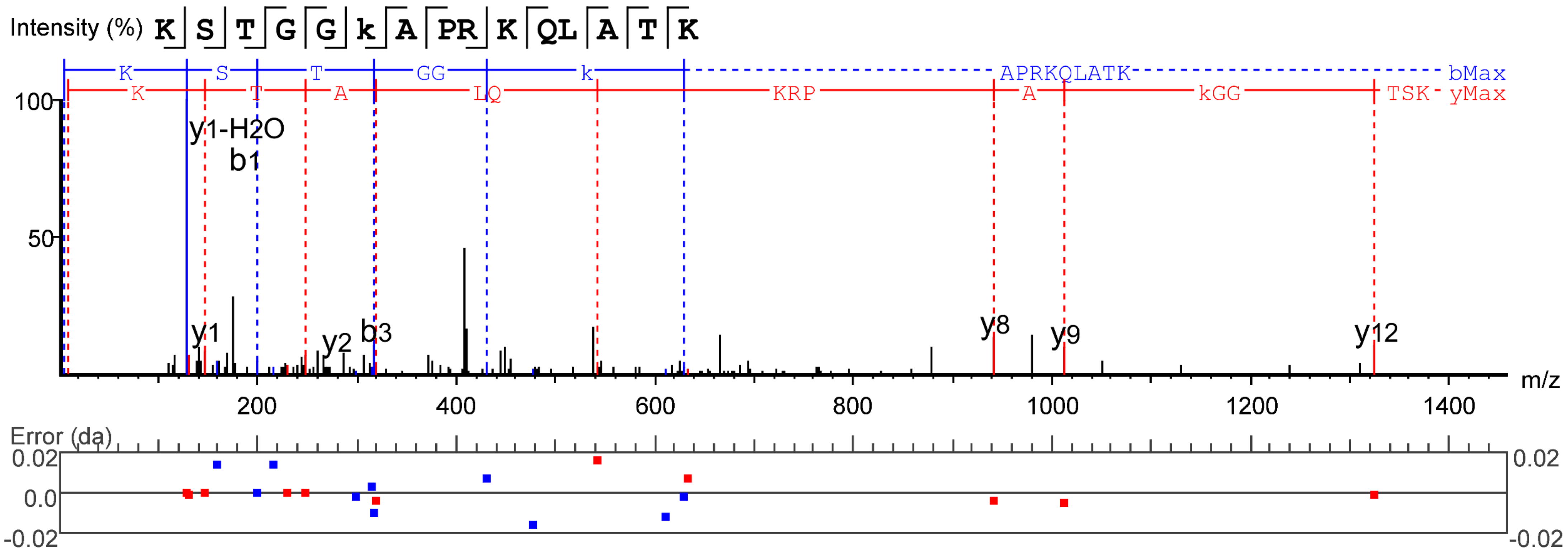

Butyrylation

KSTGGK(+70.04)APRKQLATK

Histone H3 Lys-14

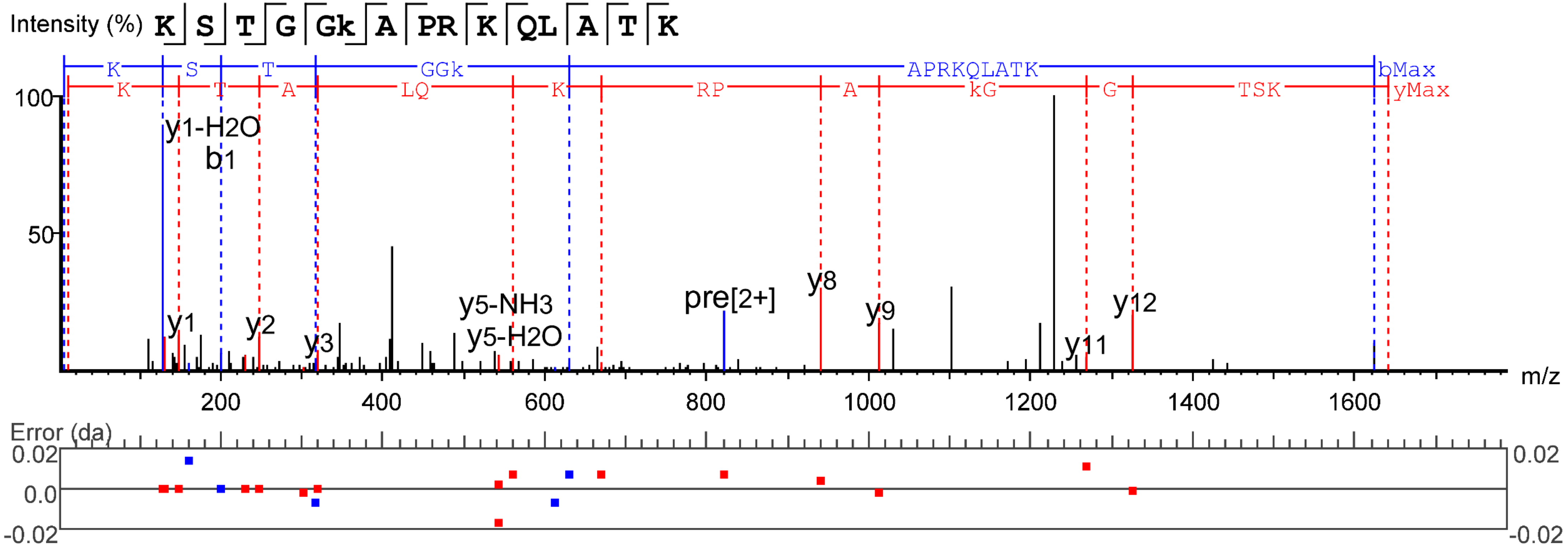

Butyrylation

GTKAVTK(+70.04)YTSSK

Histone H2B Lys-120

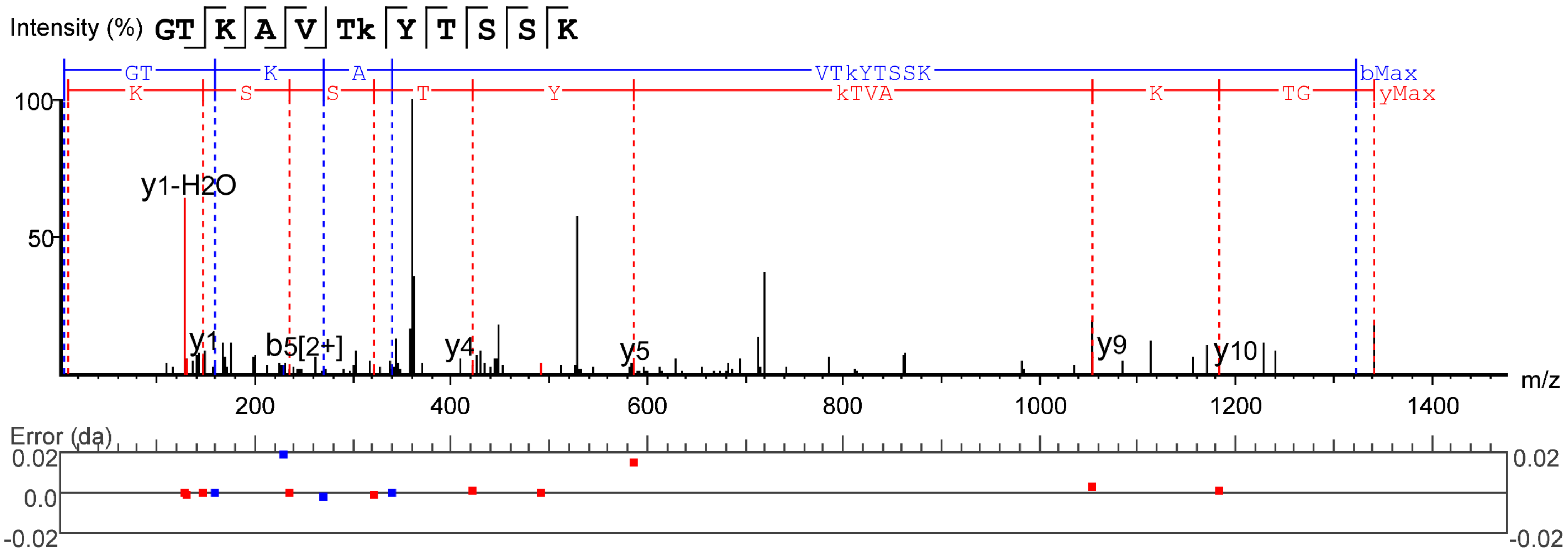

Butyrylation

KQLATK(+70.04)AAR

Histone H3 Lys-23

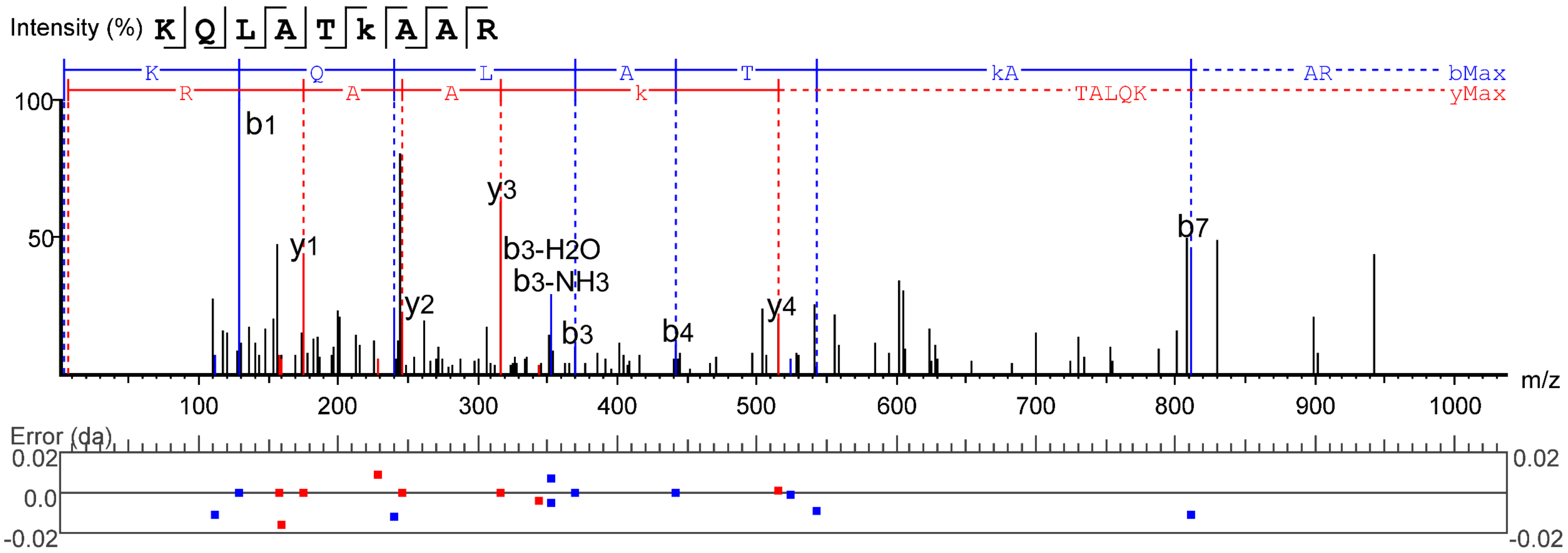

Supplement: Supplementary 2 — Mass Spectrums [file research.0109.f2.pdf]
