# Supplementary material for: Molecular Basis of KAT2A Selecting Acyl-CoA Cofactors for Histone Modifications
Source: Research (Wash D C). 2023 Apr 4;6:0109. doi: 10.34133/research.0109 (PMC10076270; doi:10.34133/research.0109)
Supplement: Supplementary 3 — Table S1. Crystallographic statistics of co-crystal structures. [file research.0109.f3.docx]

|  | propionyl-CoA complex | butyryl-CoA complex | malonyl-CoA complex | glutaryl-CoA complex |
| --- | --- | --- | --- | --- |
| **Data collection** |  |  |  |  |
| Space group  Cell dimensions | P213 | P213 | P213 | P213 |
| a, b, c (Å) | 176.182,  176.182,  176.182 | 175.695, 175.695, 175.695 | 175.692, 175.692, 175.692 | 175.265, 175.265, 175.265 |
| α, β, γ (°)  Resolution (Å) | 90, 90, 90  50-2.8  (2.85-2.8) | 90, 90, 90  50-3.0  (3.05-3.0) | 90, 90, 90  50-2.5  (2.54-2.5) | 90, 90, 90  50-3.25  (3.31-3.25) |
| R_merge_  *I/σ*(*I*) | 0.105 (0.471)  41.5 (7) | 0.114 (0.468)  11.2 (2.1) | 0.102 (0.783)  39.1 (4) | 0.139 (0.556)  11.2 (2.5) |
| Completeness (%)  Redundancy | 100 (100)  20.4 (20.9) | 98.3 (98.8)  3.7 (3.8) | 100 (100)  18.8 (18.8) | 100 (100)  7.5 (7.6) |
| **Refinement** |  |  |  |  |
| Resolution (Å)  Unique reflections  R_work_/R_free_ | 48.86-2.80  (2.91-2.80)  44545 (4422)  0.186 /0.241 | 48.73-3.00 (3.11-3.00)  35518 (3483)  0.185 / 0.255 | 43.92-2.50 (2.59-2.50)  61767 (6047)  0.192 / 0.238 | 48.61-3.26 (3.37-3.26)  28101 (2764)  0.176 / 0.236 |
| No. atoms  Protein  Ligand/ion  (specify/describe)  Water  B factors  Protein  Ligand/ion  Water  R.m.s. deviations  　Bond length (Å) | 10617  416  (propionyl-CoA)  N/A  46.34  54.70  N/A  0.009 | 10688  424  (butyryl-CoA)  N/A  46.84  60.76  N/A  0.009 | 10655  426  (malonyl-CoA)  232  49.67  57.26  47.67  0.008 | 10634  448  (glutaryl-CoA)  N/A  46.48  59.62  N/A  0.010 |
| Bond angles (°)  Ramachandran outliers (%)  Rotamer　outliers (%) | 0.91  0  2.19 | 1.09  0  0.09 | 0.88  0  0.96 | 1.17  0  0.18 |

**Supplementary Table 1. Crystallographic Statistics of Co-crystal Structures**
